# Functional Outcome after Direct Anterior Approach Total Hip Arthroplasty (DAA-THA) for Coxa Profunda and Protrusio Acetabuli—A Retrospective Study

**DOI:** 10.3390/jcm13164596

**Published:** 2024-08-06

**Authors:** Tizian Heinz, Hristo Vasilev, Philip Mark Anderson, Ioannis Stratos, Axel Jakuscheit, Konstantin Horas, Boris Michael Holzapfel, Maximilian Rudert, Manuel Weißenberger

**Affiliations:** 1 Department of Orthopaedic Surgery, University of Wuerzburg, Koenig-Ludwig-Haus, Brettreichstr 11, 97074 Wuerzburg, Germany; t-heinz.klh@uni-wuerzburg.de (T.H.); hrs.vasilev@gmail.com (H.V.); p-anderson.klh@uni-wuerzburg.de (P.M.A.); i-stratos.klh@uni-wuerzburg.de (I.S.); a-jakuscheit.klh@uni-wuerzburg.de (A.J.); k-horas.klh@uni-wuerzburg.de (K.H.); m-rudert.klh@uni-wuerzburg.de (M.R.); 2 Department of Orthopedics and Trauma Surgery, Musculoskeletal University Center, Munich (MUM), University Hospital, LMU Munich, Marchioninistr 15, 81377 Munich, Germany; boris.holzapfel@med.uni-muenchen.de

**Keywords:** total hip arthroplasty, direct anterior approach (DAA), coxa profunda, protrusio acetabuli

## Abstract

**Objective:** The direct anterior approach (DAA) is a recognized technique for total hip arthroplasty (THA) that spares soft tissue. Functional and clinical outcomes following THA via the DAA in patients with complex acetabular deformities, specifically coxa profunda (CP) and protrusio acetabuli (PA), have yet to be determined. **Methods**: A retrospective analysis was conducted on 188 primary THA cases, including 100 CP hips and 88 PA hips, performed via the DAA. Functional and clinical outcomes were evaluated by means of the Western Ontario and McMaster Universities Arthritis Index (WOMAC) and Harris Hip Score (HHS) preoperatively and at a mean follow-up of 46 ± 14 months. Furthermore, potential complications were assessed. **Results**: From the preoperative to the latest postoperative visit, a significant improvement in the WOMAC total score was observed (CP: −34.89 ± 20.66; PA: −40.38 ± 21.11). The length of stay (LOS) was the only parameter predictive of the postoperative WOMAC total score, with each day of LOS increasing the postoperative WOMAC by a mean of 1.77 points (*p* < 0.01). The HHS improved by 38.37 ± 14.23 (PA-group) and 32.79 ± 14.89 points (CP-group). No significant difference in the patient-reported outcome measures (PROMs) between the CP- and PA-group was found. The survival rate for any revision was 97.70% (PA-group) and 92.80% (CP-group). **Conclusion**: The results of this study indicate that the minimally invasive DAA was not predictive of the functional and clinical outcome following DAA-THA in patients with CP and PA. Improvements in the mean WOMAC and HHS scores were above or within the reported MCID. Additionally, revision rates were well below those reported in the literature for short and intermediate follow-up periods.

## 1. Introduction

Total hip arthroplasty (THA) has become the preferred treatment for symptomatic and advanced osteoarthritis (OA) of the hip joint, alleviating pain and restoring pain-free joint function. Due to its high success rate, THA is often referred to as the surgery of the century [1].

Despite the highly standardized surgical procedure, anatomical abnormalities such as dysplasia, coxa profunda (CP), and protrusio acetabuli (PA) pose significant challenges to the surgeon and may hamper the postoperative outcome if not adequately addressed prior to surgery. CP describes an extensively deep socket, with the floor of the acetabula fossa medial to the ilioischial line on a.p. hip radiographs [2]. In contrast, the more severe form, protrusio acetabuli (PA), is defined by medialization or protrusion of the femoral head into the acetabular fossa which can be seen on a.p. hip radiographs as the medial aspect of the femoral head lying medial to the ilioischial line [2,3,4]. Thus, PA is associated with a defect or insufficiency of the medial acetabular wall, allowing for the medial protrusion of the femoral head into the acetabular fossa and lesser pelvis. The deficient medial wall and compromised bone of the acetabular rim may yield significant intraoperative challenges that need to be anticipated during the templating and planning process prior to surgery [5,6,7]. In addition, CP and PA are often associated with a varus neck-shaft angle, which often leads to incarceration of the femoral head into the acetabular fossa, rendering femoral neck osteotomy during arthroplasty rather difficult [8]. Whilst the underlying causes of CP and PA are mostly unknown, up to 5% are reported to develop secondary to rheumatoid arthritis [6,7]. However, the prevalence of CP and PA in the general population is widely unexplored and thought to be less than 1% [9]. Some authors suggest a female predominance [10,11]. 

THA is the preferred treatment option for patients with CP or PA and advanced symptomatic OA of the hip joint. However, reconstruction of the native hip biomechanics warrants special attention in CP and PA because of the associated medialization and proximalization of the hip joint center. Various techniques have been reported for addressing the medial defect of the acetabular wall, with procedures including acetabuloplasty with morselized allograft or autograft harvested from the resected femoral head in conjunction with cemented and cementless cups [12,13,14,15]. However, the usage of bone cement alone or in conjunction with bone graft has been reported to have unsatisfactory results due to early migration and loosening of the implanted cups [12,14,15,16]. Promising results in managing THA in patients with PA have recently been reported with cementless cups, with additional autologous bone grafting if needed [8,15,17,18]. However, none of the aforementioned studies have investigated the feasibility and suitability of the minimal invasive direct anterior approach (DAA) in the treatment of PA and CP. 

Serving as the rationale of this retrospective study, the results and the potential influential factors associated with cementless cup THA in conjunction with the aspiring minimal invasive DAA for PA and CP were yet to be investigated [18,19]. It was hypothesized that the minimally invasive nature of the DAA would not be adversely associated with the typically observed performance of cementless cups in PA and CP cases. 

## 2. Materials and Methods

This cohort study was organized and reported in accordance with the STROBE (strengthening the reporting of observational studies in epidemiology, www.strobe-statement.org) checklist, ensuring a clear presentation of the conducted observational study [20]. 

### 2.1. Study Population

**Figure 1:**
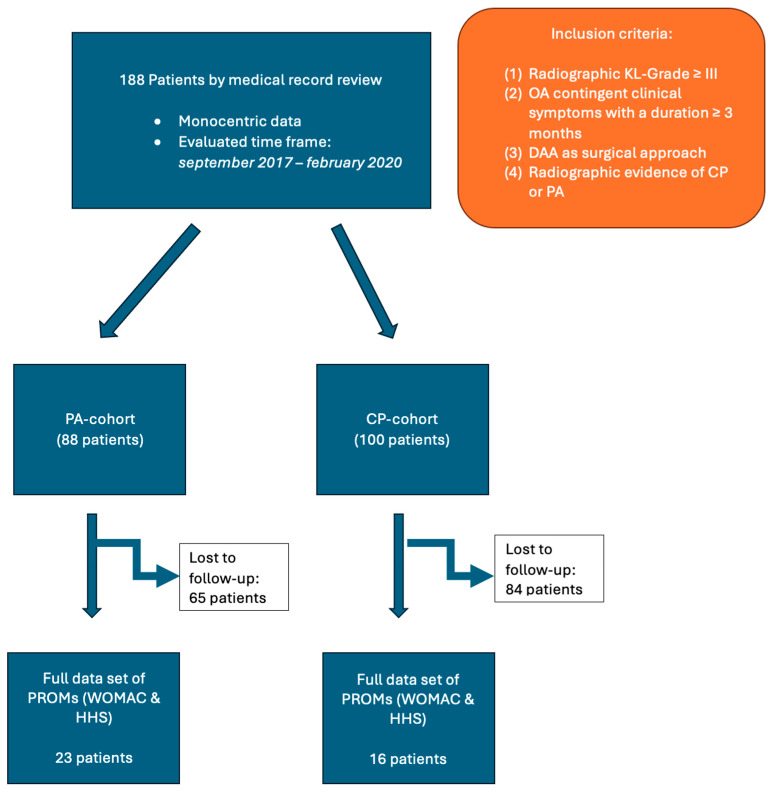
Summarizing the study design.

A cohort study design was used, and by retrospective medical record review at a single university center for orthopedic surgery in Germany, a total of 188 patients were found to be eligible for the study, as reported previously [18]. Medical records were reviewed for eligibility between September 2017 and February 2020. The inclusion criteria were based on established clinical and radiographic findings advocating and justifying the THA procedure: (1) radiographic confirmation of severe hip osteoarthritis, at least Kellgren–Lawrence grade III [21], and (2) hip osteoarthritis-contingent symptoms with ongoing pain, compromised joint function, and reduced walking distance [21,22]. Specifically, as per the primary intention of this study, the (3) inclusion of the DAA as the only surgical approach for THA was mandatory, as well as (4) radiographic evidence of CP or PA. After radiographic review, 88 and 100 patients were assigned to the PA-group and CP-group, respectively. Clinical outcomes were assessed using established patient reported outcome measurers (PROMs) that were routinely evaluated at specific time points related to the arthroplasty procedure (Table 1). However, only patients with a complete set of pre- and postoperatively available PROMs were evaluated, accounting for 23 and 16 patients in the PA- and CP-group, respectively (Figure 1). PROMs were defined as the primary outcome parameter. The severity of medial acetabular wall insufficiency (CP or in more severe cases PA) was thought to be a predictor of the surgical outcome parameters. In an attempt to reduce potential bias, clearly defined inclusion and exclusion criteria were used throughout the study. Furthermore, standardized forms and procedures for data collection were used to ensure consistency and systematic evaluation of potentially relevant parameters, thereby reducing the risk of confounding variables. Recall bias was addressed by reporting only on evaluated and well-established PROMs. Inclusion and exclusion criteria were not modified during the study, thereby addressing selection bias. Surgical complications and all readmissions related to the index procedure were evaluated. Mean follow-up was 3.84 years. This study was submitted to and approved by the local Ethics Committee (Nr. 20200619 01), ensuring accordance with the Declaration of Helsinki [23].

### 2.2. Surgical Technique

This study used the widely practiced surgical approach known as the direct anterior approach (DAA) [18,19]. To outline the procedure, patients were positioned supine on a standard operating table, and landmarks such as the greater trochanter (GT) and the anterior superior iliac spine (ASIS) were marked out prior to incision. Approximately 3 cm distal and lateral to the ASIS, the starting point for the incision could be found [24]. The incision was then extended about 5 to 6 cm distally in the direction of the lateral distal femoral condyle and the head of the fibula. Then, the fascia overlying the tensor fasciae latae (TFL) was exposed and dissected along its fibers, revealing the Hueter interval between the sartorius muscle and the TFL [24]. Subsequently, the branches of the lateral circumflex femoral vessels were ligated, followed by the femoral neck osteotomy for removal of the femoral head. Abductor tenotomy was commonly avoided by obtaining deep muscle relaxation under general anesthesia prior to head removal. Autologous morselized bone chips from the resected femoral head were utilized for augmentation in cases of extensive medial wall defects [7]. The cup was cemented in cases where there was limited stability or severe osteoporosis. After placement of the cup and liner, insertion of a bone hook into the proximal femoral canal while bringing the limb in hyperextension, adduction, and external rotation gave sufficient access for subsequent broaching. Visualization of the femoral canal was additionally improved by releasing the posterior capsule and placing a Mueller retractor under the GT [24]. The femoral canal was broached until a press fit and rotational stability was reached. The hip joint was then tested with the trial implant. Once adequate joint stability and acceptable leg length discrepancy (LLD) were achieved, the trial implant was replaced with the permanent implant of the same size, followed by intraoperative fluoroscopic verification [24]. Prior to wound closure, 2 g of tranexamic acid was injected into the hip joint. All procedures were performed by seven senior surgeons (J.A., B.H., S.B., M.R., R.S., M.W., and M.L.) using identical sets of surgical instruments. Zimmer Biomet’s ML-Taper femoral prosthesis and Allofit S Alloclassic acetabular cup were used consistently throughout the procedures.

### 2.3. Radiographic Features

Pelvic radiographs were taken using a standardized procedure and meticulously checked for tilt or rotation discrepancies before templating and measurement. These images were digitally archived using the Picture Archiving and Communication System (PACS), and measurements were performed using the angle and measurement tools available in the X-ray viewer (DeepUnity Review, DH Healthcare GmbH, Bonn, Germany). All measurements were performed on plain standing anteroposterior pelvic radiographs with 15 degrees of internal rotation [2].

Radiomorphologic features were used to differentiate between CP and PA: CP was identified when the medial wall of the acetabular fossa was medial to the ilioischial line while the medial cortex of the femoral head remained lateral or aligned with the ilioischial line. Conversely, PA was identified when both the medial wall of the acetabular fossa and the medial cortex of the femoral head were medial to the ilioischial line. In addition, the degree of PA was further delineated by measuring the horizontal distance between the ilioischial line (also referred to as the Kohler line) and the medial acetabular margin, referred to as the AK distance. After surgery, the medial edge of the acetabular component served as a substitute for the medial acetabular wall. Based on the AK distance, gradations were established: (1) 1 to 5 mm was indicative of mild PA, (2) 6 to 15 mm was indicative of moderate PA, and (3) AK distance greater than 16 mm was indicative of severe PA [19]. A detailed radiographic analysis of this patient cohort has been described elsewhere [18].

### 2.4. Statistical Analysis

Data analysis was conducted using SPSS software (version 27, SPSS Inc., Chicago, IL, USA). Ordinal variables were presented as means with standard deviations, while categorical variables were described using absolute and relative frequencies. The Kolmogorov–Smirnov test was employed to assess the normality of data distribution. Group differences between CP and PA were analyzed using either the independent *t*-test or the Mann–Whitney U test. Categorical variable frequencies were compared using the chi-square test. Within-group differences over time (preoperative to postoperative) were evaluated using the dependent *t*-test or Wilcoxon test. Additionally, logistic and linear regression analyses were performed to investigate the impact of several independent factors on the outcome variables, thereby adjusting for confounding variables. A priori sample size calculation was performed using G-power (version 3.1) [25], assuming a conservative effect size and a statistical power of 0.8, which translated to a total sample size of 38 patients. A significance threshold was set at *p* < 0.05.

## 3. Results

### 3.1. Patient Demographics

A total of 188 patients were included in the study cohort, with 88 and 100 patients being assigned to the PA-group and CP-group, respectively. A strong predominance of female patients was found in both cohorts. The mean age was significantly higher in the PA-group compared to patients with coxa profunda. A significant correlation was found between hip morphology (protrusio acetabuli and coxa profunda) and the type of THA fixation, with CP patients having a higher likelihood of cementless fixation (*p* = 0.01). Patient demographics and characteristics are shown in Table 2. 

### 3.2. Radiographic Outcome 

When stratified according to the AK distance, 33 and 50 patients were identified with mild and moderate PA. Moreover, five cases with severe PA defined by an AK distance greater than 16 mm were found. Postoperatively, the PA was fully treated in 59 cases (67.05%) by transferring the medial border of the acetabular component lateral to or flush with the ilioischial line. In the remaining 29 cases, the PA was not fully treated, but the AK distance was reduced by a mean of 3.93 ± 4.53 mm. A more detailed radiographic analysis of this study cohort has been previously reported [18]. 

### 3.3. Clinical Outcome and PROMs

**Figure 2:**
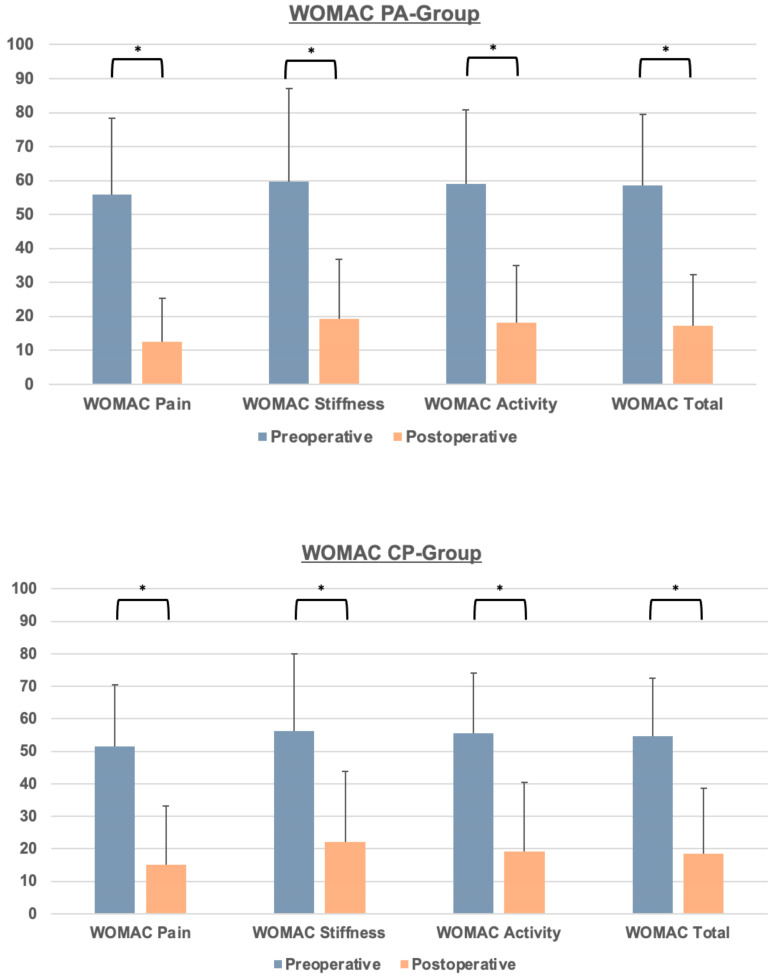
Pre- and postoperative WOMAC scores for the PA- and CP-groups. Significant differences (*p* < 0.05) are marked by asterisks.

A significant decrease in the WOMAC total score and WOMAC subscores were observed in both the CP- and PA-groups from the preoperative to the postoperative visit (Figure 2). The mean improvement for the CP- and PA-groups at the last follow-up visit was −34.89 ± 20.66 and −40.38 ± 21.11, respectively (Table 3). Prior to surgery, there were no statistically significant differences in the WOMAC total score or its subscores between the CP- and PA-groups. The observed improvement was similar across both groups, with no statistically significant differences in WOMAC total and subscores at the last follow-up (Table 3). The length of stay (LOS) was the only parameter predictive for the postoperative WOMAC total score, with every day of LOS increasing the postoperative WOMAC by a mean of 1.77 points (*p* < 0.01). 

**Figure 3:**
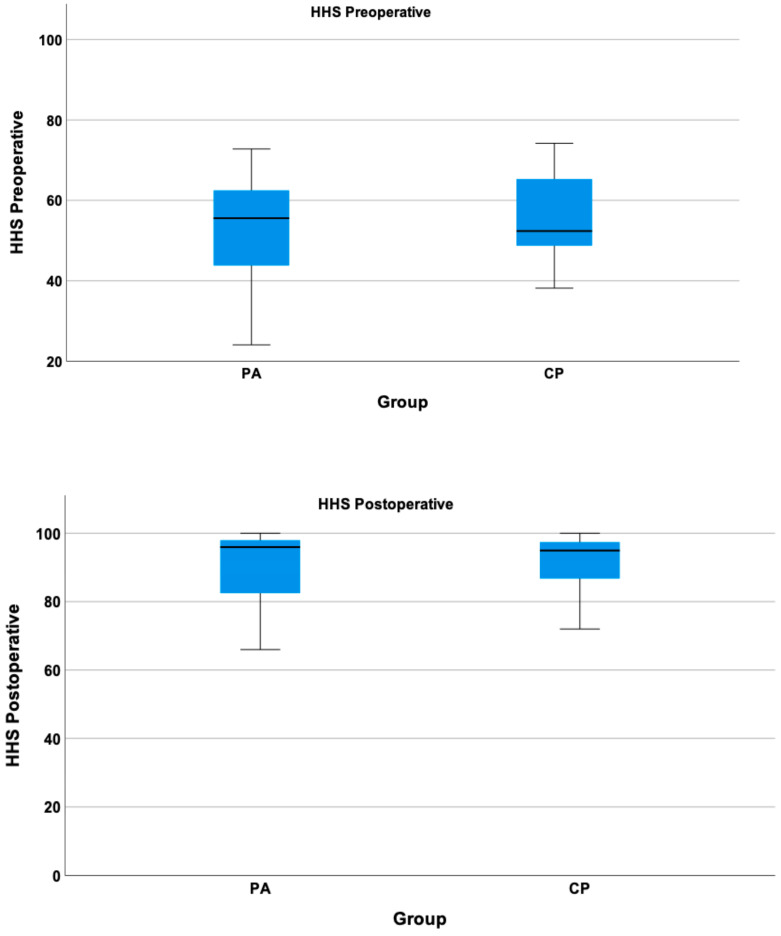
Pre- and postoperative HHS scores for the CP- and PA-group.

Regarding the HHS, patients with PA had a lower mean HHS compared to patients with CP at the preoperative visit, though it was not statistically significant (Figure 3). Both the CP- and PA-group showed a significant improvement from the preoperative to the postoperative visit (*p* < 0.01), with mean improvements in the HHS of 38.37 ± 14.23 (PA-group) and 32.79 ± 14.89 (CP-group), respectively. At the last follow-up, values of the HHS were not statistically different between the CP- and PA-group. 

The LOS, ASA, and change in hemoglobin (Delta HB) were the only parameters showing a significant correlative association with postoperative HHS. 

**Figure 4:**
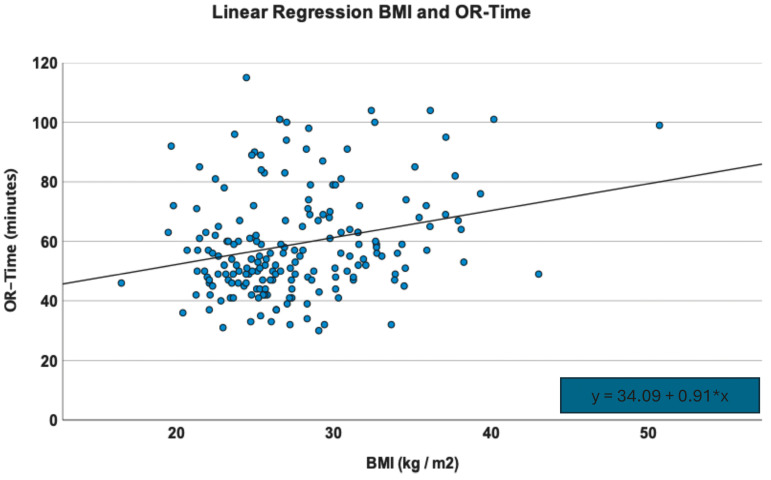
Correlative association of the BMI and the OR-time by linear regression analysis.

Furthermore, there was a statistically significant linear association of the BMI and the duration of surgery, with every increase in the BMI by one unit leading to an elevated surgery time of 0.91 min (R^2^ = 0.06, F(1) = 12.57, *p* = 0.01) (Figure 4). However, duration of surgery was not significantly different in both groups, with a mean OR-time of 59.10 ± 17.75 min. 

The mean hemoglobin drop from the preoperative to the postoperative visit (3 days after surgery) turned out to be 2.79 ± 1.14 g/dL, without any significant difference in both groups. 

Intraoperative and postoperative complications, such as anemia, respiratory infection, prolonged wound healing, or nerve injury, showed no significant differences between the two groups (Table 4). In the PA-group, two patients required revision surgery at 3 and 4 weeks postoperatively due to acetabular cup loosening (one case) and superficial wound infection (one case). In the CP-group, one patient underwent revision of the femoral component 4 weeks postoperatively due to a periprosthetic fracture. Additionally, two patients in the CP-group were readmitted at 8 weeks and another patient at 2 years, all due to periprosthetic infection. The mean follow-up period screened for readmission at the index hospital was 50.13 months. Survival analysis showed no significant difference for the PA- and CP-group, with a survival rate of 97.70% and 92.80% during the mean follow-up period for the PA- and CP-group for any revision as endpoint. With acetabular loosening as the endpoint, the survival rate in the CP-group and PA-group was 100% and 98.90%, respectively. 

## 4. Discussion

PA and CP, though relatively rare, represent a complex hip morphology, rendering primary hip arthroplasty a complex surgical procedure. It was the primary intention of this study to investigate whether complex hip deformities such as CP and PA are still associated with favorable PROMs when undergoing arthroplasty through the minimally invasive DAA. With the recent extension of the DAA to hip revision cases, it was hypothesized that complex primary hip deformities like CP and PA would not be influenced by the minimally invasive nature of the DAA. Furthermore, the authors have recently demonstrated promising radiographic results following primary THA in CP and PA cases [18], but the relation with clinical outcome data was still lacking. 

As a main finding of this study, cementless cup THA performed through the minimally invasive DAA was associated with satisfying and promising patient-reported outcome measures in the cases of both PA and CP. Specifically, in the CP-group and PA-group, the mean increase in the HHS from preoperative to the last visit turned out to be 32.79 ± 14.89 and 38.37 ± 14.23 points. Singh et al. reported a minimal clinically important difference (MCID) for the HHS of 18.0 and 15.9 points following two and five years from the arthroplasty procedure [26]. With the HHS change score of this study cohort being significantly higher than the reported MCID, a clinically important gain in function and pain was inferred. Similarly, the mean change score for WOMAC Pain turned out to be well within the reported MCID for both groups [27]. Regarding the WOMAC Function subscore, the mean change scores (CP: 35.18 ± 21.86; PA: 40.12 ± 21.30) were well within the reported MCID [27]. Therefore, based on the results of the HHS and WOMAC scores, a remarkably high gain in function and pain was observed in both groups after hip arthroplasty with the DAA.

Traditionally, cementation of the acetabular component for the management of PA has been suggested for a while. The idea behind this outdated recommendation was that the bone cement would aid in supporting and bridging the deficient medial wall and facilitate placement of the cup in a more anatomical position [14,17,28,29]. However, the mid- and long-term data showed a remarkably high rate of aseptic acetabular loosening and recurrence of acetabular protrusion in those cases treated with cementation of the acetabular component, leading to a paradigm shift towards the use of cementless cups with or without autologous bone support, demonstrating promising results [7,30,31]. Similarly, Baghdadi et al. demonstrated a considerably higher mid- and long-term survival rate of the socket when using a non-cemented porous coated cup compared to a cemented cup [8,32]. Regardless of the fixation technique, meticulous reconstruction of the center of the native rotation (COR), which is usually moved superiorly and medially due to the medial acetabular defect, has been identified as a major predictor determining the survival rate of the acetabular component. Thus, a 24% risk of aseptic cup loosening has been associated with each 1 mm of undercorrection of the native COR [32,33]. Reported survival rates of the implant following THA range from 80% to 90% for short and intermediate follow-up periods [33,34,35]. With a survival rate of 97.8% in this study cohort, a slightly better mean survival for any surgical revision than what is commonly reported in the literature was found. This finding is also supported by the satisfying and promising radiographic reconstruction parameters achieved in this study cohort [18]. 

Another noteworthy finding of this study was that the clinical outcome data between the CP- and PA-group did not have a statistically significant difference. Since CP is generally considered a less complicated anatomical variant compared to PA, this finding suggests that the surgical approach to the hip joint may not significantly influence the outcomes of complex hip arthroplasty. 

To the knowledge of the authors, this is the first study reporting on the DAA for the management of CP and PA on a large patient cohort. Recently, the DAA has experienced an unprecedented rise worldwide due to its minimally invasive and tissue-sparing nature [36]. This has led to a gradual expansion of the DAA to more complex primary THA cases such as developmental dysplasia of the hip (DDH), and revision arthroplasty via the DAA has also recently been reported [37,38]. The main idea behind this trend is to transfer the potential merits of the DAA to complex primary THA, namely early postoperative mobilization, less intraoperative blood loss, and decreased dislocation risk [39,40]. 

In conclusion, the DAA seems to not constitute a limiting factor in the management of OA in patients with medial acetabular protrusion. The large increase in common PROMs demonstrates a significant gain in hip function and decrease in hip pain in the study cohort. This sharp improvement of the WOMAC and HHS scores during the follow-up period may be partially due to the minimally invasive nature of the DAA. Meanwhile, several studies have exhibited a significant benefit of the DAA at the short and intermediate follow-up [40,41,42]. Furthermore, the uncomplicated implementation of fluoroscopy in conjunction with the DAA facilitates intraoperative control of cup placement and eases restoring of the COR.

It is noteworthy, given the retrospective nature of this study, that there are inevitably some shortcomings: firstly, the lack of a control group limits the generalizability of the results from this study cohort. Secondly, an extension of the follow-up period to at least ten years would have aided in examining the long-term fate and revision rates of the DAA-THA. Notably, there was a strong predominance of female patients, which may cause potential bias. However, CP and PA are known to have a strong female predominance, and multivariate regression analysis did not find gender to be a potentially confounding variable. 

This study is, to our knowledge, the first to explore the clinical outcome of the DAA for complex THA in CP and PA patients. The substantial sample size of 100 hips in the PA-group increases the robustness of the results. Therefore, the findings of the present study may serve as a starting point for future research on this topic, and prospective study designs will be needed to finally evaluate the safety and efficacy of the DAA in conjunction with complex PA and CP hip arthroplasty. 

## 5. Conclusions

The results of this study indicate that the minimally invasive DAA was not predictive of the functional and clinical outcome following DAA-THA in patients with CP and PA. Improvements in the mean WOMAC and HHS scores were above or within the reported MCID. Additionally, revision rates were well below those reported in the literature for short and intermediate follow-up periods.

## Figures and Tables

**Table 1:** Type and timing of PROMs during the study period.

Type of PROM	Time of Evaluation
EQ5D	Preoperative 1 year postoperative 5 years postoperative
WOMAC	Preoperative 1 year postoperative 5 years postoperative
Harris Hip Score (HHS)	Preoperative 3 years postoperative
VAS	Preoperative 1 year postoperative

**Table 2:** Patient demographics and characteristics.

Item	PA-Group	CP-Group	
	Mean (±SD), rel. frequency		*p*-value (CP-group vs. PA-group)
Age (years ± SD)	71.61 ± 12.41	67.05 ± 10.23	0.01
ASA	I: 1 (1.1%) II: 56 (63.6%) III: 30 (34.1%) IV: 1 (1.1%)	I: 6 (6.0%) II: 65 (65.0%) III: 28 (28.0%) IV: 1 (1.0%)	0.31
BMI (kg/m^2^)	27.24 ± 4.61	27.91 ± 5.25	0.36
Sex (female/male)	75/13, 85.20%/14.80%	82/18, 82.0%/18.0%	0.35
Hip flexion preoperative (degrees)	82. 63 ± 17.80	90.20 ± 16.60	0.00
Hip flexion postoperative (degrees)	112.14 ± 9.17	101.67 ± 20.05	0.02
Method of fixation (cementless/hybrid/fully cemented)	72/11/5	94/6/0	0.01
LOS (days ± SD)	9.11 ± 3.58	8.44 ± 2.10	0.11

**Table 3:** Pre- and postoperative WOMAC scores for the CP- and PA-group. Significances for within-group changes and between-group changes are given.

	CP-Group	PA-Group	*p*-Value (CP-Group vs. PA-Group)
WOMAC Pain preoperative	51.60 ± 19.00	55.86 + 22.39	0.17
WOMAC Pain postoperative	15.08 ± 18.06	12.60 ± 12.79	0.36
*p*-value (preoperative vs. postoperative)	<0.00	<0.00	
WOMAC Stiffness preoperative	56.16 ± 23.83	59.73 ± 19.21	0.35
WOMAC Stiffness postoperative	22.10 ± 21.70	19.21 ± 17.51	0.39
*p*-value (preoperative vs. postoperative)	<0.00	<0.00	
WOMAC Activity preoperative	55.61 ± 18.45	58.94 ± 18.12	0.26
WOMAC Activity postoperative	19.18 ± 21.30	18.16 ± 16.81	0.76
*p*-value (preoperative vs. postoperative)	<0.00	<0.00	
WOMAC Total preoperative	54.65 ± 17.83	58.52 ± 20.94	0.18
WOMAC Total postoperative	18.58 ± 20.11	17.15 ± 15.07	0.64
*p*-value (preoperative vs. postoperative)	<0.00	<0.00	

**Table 4:** Complication rates of the CP- and PA-group during the follow-up period of a mean of 50.13 months.

	CP-Group	PA-Group
Complication rates (total number n, percent %)		
Postoperative anemia	3 (3.00%)	2 (2.27%)
Prolonged wound healing	4 (4.00%)	4 (4.54%)
Postoperative regional paresthesia	2 (2.00%)	1 (1.14%)
Respiratory infection	2 (2.00%)	2 (2.27%)
Intraoperative fracture (femur or acetabulum)	1 (0.53%)	2 (1.06%)

## Data Availability

Data can be obtained from the authors upon reasonable request.
